# Supplementary material for: Preparation of Hybrid Nanopigments with Excellent Environmental Stability, Antibacterial and Antioxidant Properties Based on Monascus Red and Sepiolite by One-Step Grinding Process
Source: Nanomaterials (Basel). 2023 Jun 2;13(11):1792. doi: 10.3390/nano13111792 (PMC10254824; doi:10.3390/nano13111792)
Supplement: Supplementary file 1 [file nanomaterials-13-01792-s001.zip › nanomaterials-2414252-SI.pdf]

Preparation of Hybrid Nanopigments with Excellent Environmental Stability, Antibacterial and Antioxidant Properties Based on *Monascus* Red and Sepiolite by One-Step Grinding Process

Shue Li [1,2], Penji Yan [3], Bin Mu [1,*], Yuru Kang [1], Aiqin Wang [1,*]

1 Key Laboratory of Clay Mineral Applied Research of Gansu Province, Center of Eco-Material and Green Chemistry, Lanzhou Institute of Chemical Physics, Chinese Academy of Sciences, Lanzhou 730000, China;seli17@licp.cas.cn (S.L.); yurukang@licp.cas.cn (Y.K.)
2 Center of Materials Science and Optoelectronics Engineering, University of Chinese Academy of Sciences, Beijing 100049, China
3 College of Chemistry and Chemical Engineering, Key Laboratory of Hexi Corridor Resources Utilization of Gansu Universities, Hexi University, Zhangye 734000, China; yanpenji@hxu.edu.cn (P.Y.)
* Correspondence: mubin@licp.cas.cn (B.M); aqwang@licp.cas.cn (A.W.); Tel.: +86-931-4868118 (A.W.)

I: Computational details and models

Computational details and models

The calculations and molecular geometries were performed utilizing the DFT method implemented in the commercial Gaussian 09 program package and the M062X(D3) functional [1]. For all the atoms, the 6-31G* Pople basis was set at the same level as the SMD [2] solvation model. The frequency calculations at the same level were applied to appraise the stationary points as minima once the convergences of optimizations were obtained. The electrostatic potential (ESP) [3] and restrained electrostatic potential (RESP) atomic charge [4] analyses were finished via the Multiwfn 3.8(dev) code, and the ESP maps were rendered via the VMD [5] visualization program based on the files exported by Multiwfn. All of the optimized geometries mentioned were built by Gaussview 6.0 [6]. Furthermore, the PSI4 program [7] was used for zeroth-order symmetry-adapted perturbation theory (SAPT0) calculations [8].

II: Supplementary Figures

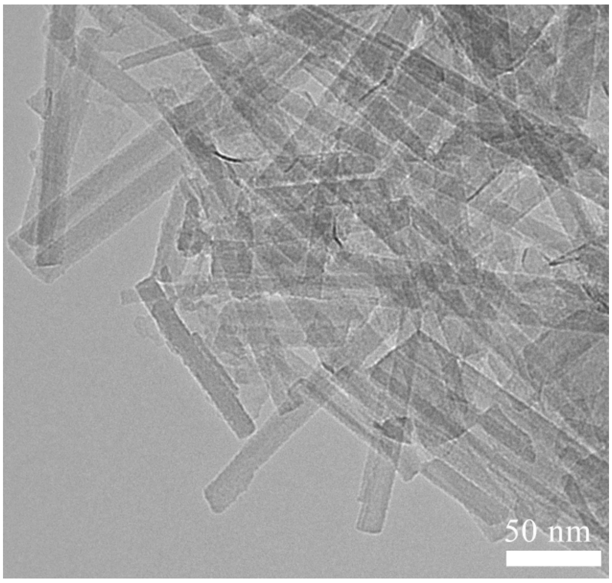

Figure S1. TEM image of DTAB/MR/Sep.

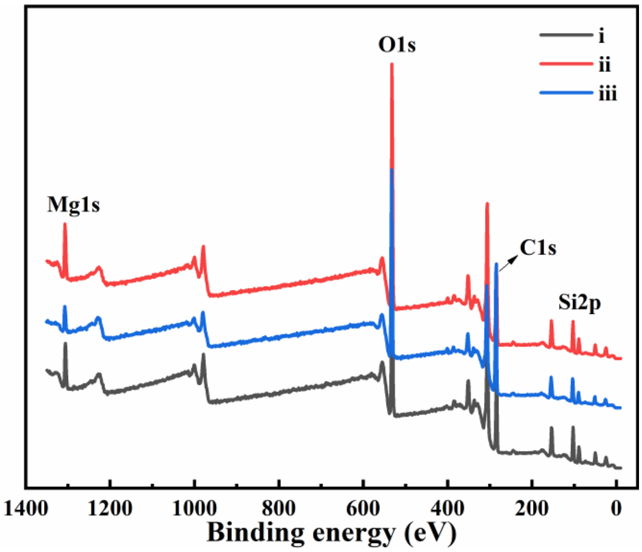

Figure S2. XPS survey spectra of i) DTAB/Sep, ii) MR/Sep, and iii) DTAB/MR/Sep.

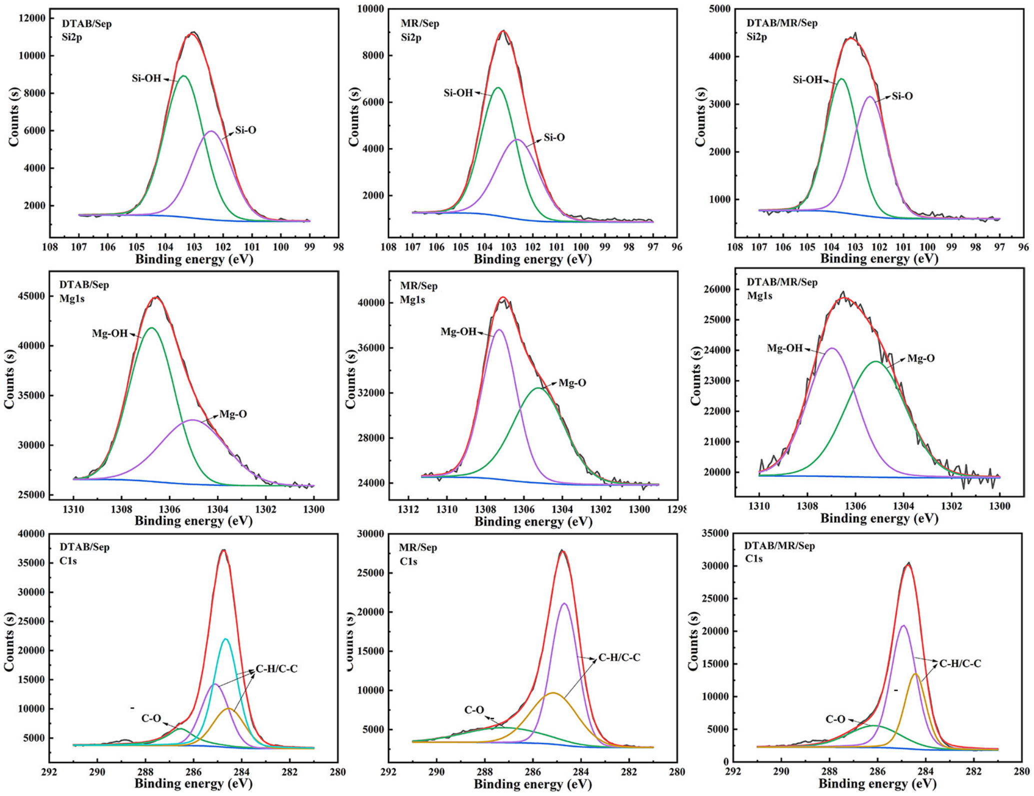

Figure S3. High-resolution XPS spectra of Si2p, Mg1s, and C1s of DTAB/Sep, MR/Sep, DTAB/MR/Sep.

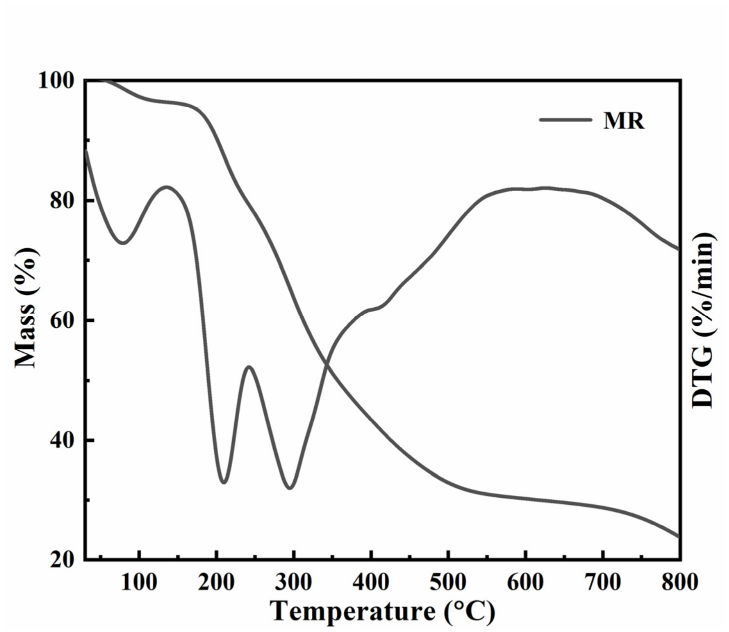

Figure S4. TGA and DTG curves of MR.

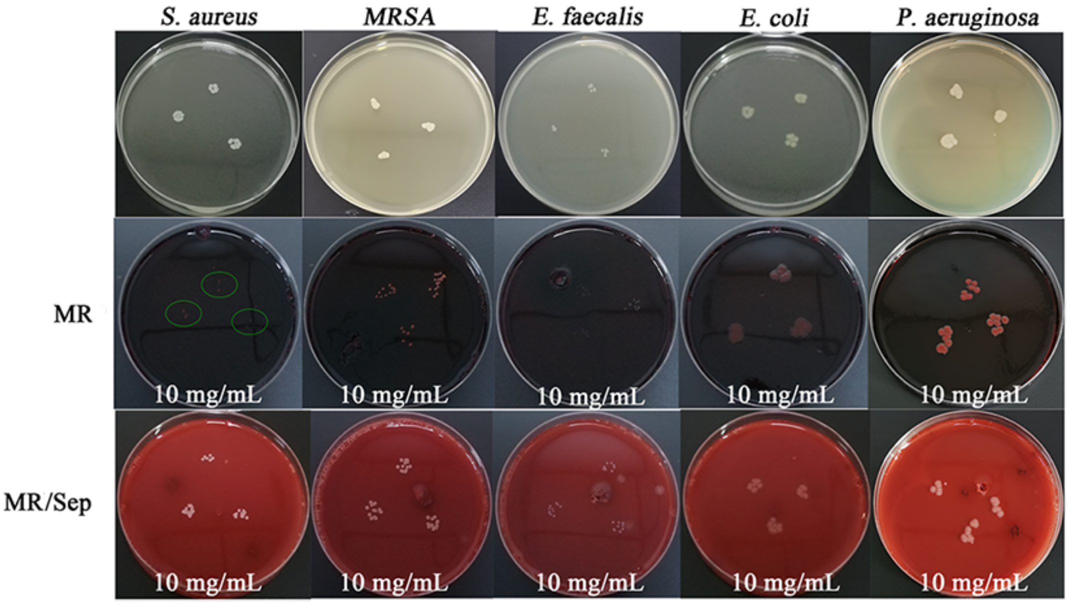

Figure S5. Positive control of *S. aureus*, *MRSA*, *E. faecalis*, *E. coli*, and *P. aeruginosa*, as well as the above bacteria treated with MR and MR/Sep.

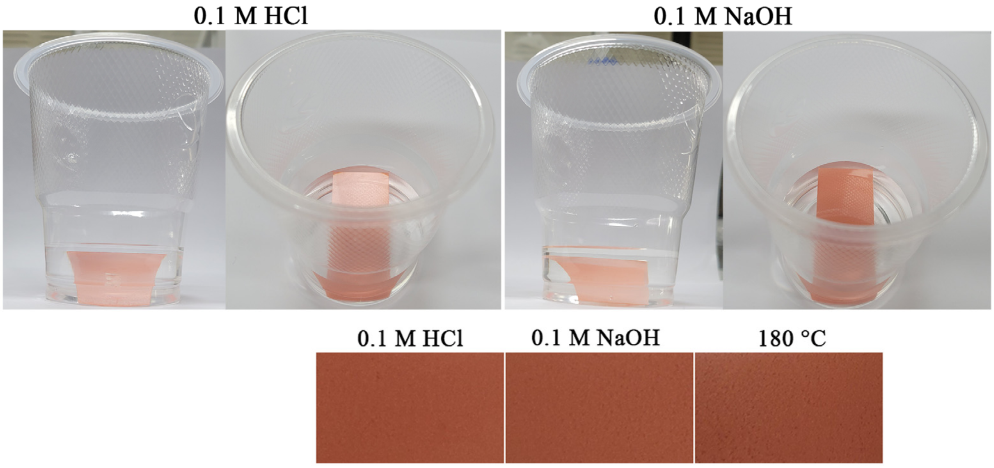

Figure S6. Digital photos of DTAB/MR/Sep@fluoroPOS coatings after treatment under various conditions for 0.5 h.

III: Supplementary Tables

Table S1. XPS chemical compositions of DTAB/Sep, MR/Sep, DTAB/MR/Sep, and DTAB/MR/Sep@fluoro POS.

Samples	At. %						
	C	O	Fe	Si	Al	Mg	F
DTAB/Sep	35.78	41.09	0.43	14.85	2.45	4.86	-
MR/Sep	36.36	41.69	0.31	13.5	1.96	5.73	-
DTAB/MR/Sep	52.35	31.55	0.34	10.63	1.39	3.22	-
DTAB/MR/Sep@fluoroPOS	27.40	32.76	1.47	12.43	1.39	3.93	20.48

Table S2 Results of SAPT(0)/jun-cc-pVDZ non-covalent interaction energy analyses for several complexes.

Stationary points	Electrostatics (kcal/mol)	Exchange (kcal/mol)	Induction (kcal/mol)	Dispersion (kcal/mol)	Total (kcal/mol)
Sep	-42.97	35.00	-11.44	-8.37	-27.78
MR/Sep-Ⅰ	-52.31	49.55	-22.09	-20.09	-44.94
MR/Sep-Ⅱ	-53.35	49.03	-19.06	-20.68	-44.07
MR/Sep-Ⅲ	-11.36	37.98	-13.06	-19.1	-5.54
MR/Sep-Ⅳ	-37.74	28.51	-13.86	-14.01	-37.09
DTAB/MR	-12.26	21.25	-5.63	-19.75	-16.06

Table S3. CIE parameters of MR, MR/Sep, DTAB/MR/Sep, and CTAB/MR/Sep.

**Samples**	**Color Parameters**			***C****	***h**°*
	L*	***a****	***b****		
MR	8.78	11.00	11.88	16.19	47.19
MR/Sep	40.93	32.29	19.02	37.47	30.51
DTAB/MR/Sep	32.55	31.44	16.80	35.65	28.12
CTAB/MR/Sep	39.17	30.09	15.44	33.82	27.17

Table S4. Minimum inhibitory concentration (MIC) (mg/mL) of hybrid nanopigments against *S. aureus*, *MRSA*, *E. faecalis*, *E. coli*, and *P. aeruginosa*.

**Samples**	**MIC values (mg/mL)**				
	S. aureus	***MRSA***	***E. faecalis***	***E. coli***	***P. Aeruginosa***
DTAB/MR/Sep	0.25	0.25	0.50	5.00	10.00
CTAB/MR/Sep	0.10	0.10	0.10	-	-

Table S5. CAs and SAs of liquid droplets on the untreated DTAB/MR/Sep@ fluoro POS coating and water droplets on the DTAB/MR/Sep@fluoro POS after treatment at various conditions for 0.5 h.

**Treatment conditions**	**Liquids**	**CA (°)**	**SA (°)**
-	Water	155.20±0.60	3.62±0.66
-	Glycerol	153.03±1.16	4.49±0.18
Heating at 180°C	Water	153.63±1.10	5.78±0.47
Immersed into 0.1 M HCl	Water	153.87±0.32	>15
Immersed into 0.1 M NaOH	Water	155.87±0.12	>15

Table S6. CAs and SAs of water droplets on the DTAB/MR/Sep@fluoro POS after two acid/base cycles.

Different atmospheres	CA (°)	SA (°)
HCl	155.30±1.23	>15
NH_3	154.57±0.90	>15

REFERENCES

1. Frisch, M.J.; Trucks, G.W.; Schlegel, H.B.; Scuseria, G.E.; Robb, M.A.; Cheeseman, J.R.; Scalmani, G.; Barone, V.; Mennucci, B.; Petersson, G.A.; Nakatsuji, H. (Gaussian 09, revision A.1; Gaussian, Inc.: Wallingford, CT, **2009**.).

2. Marenich, A.V.; Cramer, C.J.; Truhlar, D.G. Universal Solvation Model Based on Solute Electron Density and on a Continuum Model of the Solvent Defined by the Bulk Dielectric Constant and Atomic Surface Tensions. *J. Phys. Chem. B* **2009**, 113, 6378–6396.

3. Lu, T.; Chen, F.W. Quantitative analysis of molecular surface based on improved Marching Tetrahedra algorithm. *J. Mol. Graph. Model* **2012**, 38, 314-23.

4. Bayly, C.I.; Cieplak, P.; Cornell, W.D.; Kollman, P.A. A well-behaved electrostatic potential based method using charge restraints for deriving atomic charges: The RESP Model. *J. Phys. Chem.* **1993**, 97, 10269-10280.

5. Humphrey W, D.A.; Schulten K. VMD: , Visual molecular dynamics, *J. Mol. Graph. Model.* **1996**, 14, 33-38.

6. Semichem, I. GaussView 6.0.16 program: http://gaussian.com/gaussview6/ (accessed on Nov 25, 2019). Semichem, Inc., GaussView 6.0.16 program: http://gaussian.com/gaussview6/ (accessed on Nov 25, 2019).

7. Parrish, R.M.; Burns, L.A.; Smith, D.G.A.; Simmonett, A.C.; DePrince, A.E.; Hohenstein, E.G.; Bozkaya, U.; Sokolov, A.Y.; Di Remigio, R.; e. al., Psi4 1.1: An Open-Source Electronic Structure Program Emphasizing Automation, Advanced Libraries, and Interoperability, *J. Chem. Theory Comput.* **2017**, 13, 3185-3197.

8. Emamian, S.; Lu, T.; Kruse, H.; Emamian, H. Exploring Nature and Predicting Strength of Hydrogen Bonds: A Correlation Analysis Between Atoms-in-Molecules Descriptors, Binding Energies, and Energy Components of Symmetry-Adapted Perturbation Theory, *J. Comput. Chem.* **2019,** 40, 2868-2881.
